# Ferroelectric Tunneling Junctions Based on Aluminum Oxide/ Zirconium-Doped Hafnium Oxide for Neuromorphic Computing

**DOI:** 10.1038/s41598-019-56816-x

**Published:** 2019-12-31

**Authors:** Hojoon Ryu, Haonan Wu, Fubo Rao, Wenjuan Zhu

**Affiliations:** 1 Department of Electrical and Computer Engineering, University of Illinois at Urbana-Champaign, Urbana, IL 61801 USA; 2 Materials Research Laboratory, University of Illinois at Urbana-Champaign, Urbana, IL 61801 USA

**Keywords:** Electrical and electronic engineering, Nanoscale devices

## Abstract

Ferroelectric tunneling junctions (FTJs) with tunable tunneling electroresistance (TER) are promising for many emerging applications, including non-volatile memories and neurosynaptic computing. One of the key challenges in FTJs is the balance between the polarization value and the tunneling current. In order to achieve a sizable on-current, the thickness of the ferroelectric layer needs to be scaled down below 5 nm. However, the polarization in these ultra-thin ferroelectric layers is very small, which leads to a low tunneling electroresistance (TER) ratio. In this paper, we propose and demonstrate a new type of FTJ based on metal/Al_2_O_3_/Zr-doped HfO_2_/Si structure. The interfacial Al_2_O_3_ layer and silicon substrate enable sizable TERs even when the thickness of Zr-doped HfO_2_ (HZO) is above 10 nm. We found that F-N tunneling dominates at read voltages and that the polarization switching in HZO can alter the effective tunneling barrier height and tune the tunneling resistance. The FTJ synapses based on Al_2_O_3_/HZO stacks show symmetric potentiation/depression characteristics and widely tunable conductance. We also show that spike-timing-dependent plasticity (STDP) can be harnessed from HZO based FTJs. These novel FTJs will have high potential in non-volatile memories and neural network applications.

## Introduction

Recently, brain-inspired computing, or neuromorphic computing, has garnered intense research attention. Neuromorphic computing aims to realize electronic systems that emulate the computational energy-efficiency and fault tolerance of the biological brain in a compact space^1–3^. Traditional computing systems based on von Neumann architecture have severe limitations in energy consumption, deep-learning capability and scalability to large networks^4,5^. In contrast, the human brain is capable of performing much more intelligent functions and yet consumes much less power and occupies less space^3^. To bridge this gap, researchers are actively investigating the possibilities to construct electronic systems emulating the neuronal organization and function of nervous systems. In the past, a broad spectrum of devices with programmable conductance, such as phase change memories and resistive change memories, have been explored as potential synaptic devices^1,6–11^. As compared to these candidates, the ferroelectric-based synaptic devices have several advantages including high symmetry in potentiation/depression operations and fast response, which are important traits for data processing and on-chip learning^12–16^. In this paper, we investigate ferroelectric tunneling junctions (FTJs) as the synaptic devices for neuromorphic computing.

FTJ is a two-terminal device composed of a thin ferroelectric layer sandwiched in between two electrodes. The tunneling resistance in an FTJ can be switched between a high and a low value by the reversal of the polarization in the ferroelectric material. Traditionally, the FTJs are mainly based on perovskites, such as Barium titanate (BaTiO_3_) and lead zirconate titanate (PZT)^17,18^. However, these materials are incompatible with CMOS processes, and their thickness is not easily scaled. Recently, it was discovered that doped hafnium oxide (HfO_2_) has strong ferroelectricity^19–28^. As compared to traditional perovskites, doped HfO_2_ has many advantages, including excellent scalability and full compatibility with CMOS processes^28–31^. Among various doped HfO_2_, Zr-doped HfO_2_ (HZO) is particularly attractive due to its low annealing temperature and excellent scalability^25,31–34^. In FTJs based on metal/HZO/metal structure, in order to achieve a sizable on-current, the thickness of HZO needs to be scaled down below 5 nm^35,36^. However, the polarization in these ultra-thin HZO films is very small, which leads to a low TER ratio^37^. In this project, we propose and demonstrate a new type of FTJ based on metal/Al_2_O_3_/HZO/Si structure. The interfacial Al_2_O_3_ layer and semiconducting substrate enable sizable TERs even when the thickness of HZO is above 10 nm. We demonstrate FTJ synapses with symmetric potentiation/depression characteristics and widely tunable conductance. We also show that spike-timing-dependent plasticity (STDP) can be harnessed from HZO based FTJs.

## Results and Discussion

**Figure 1:**
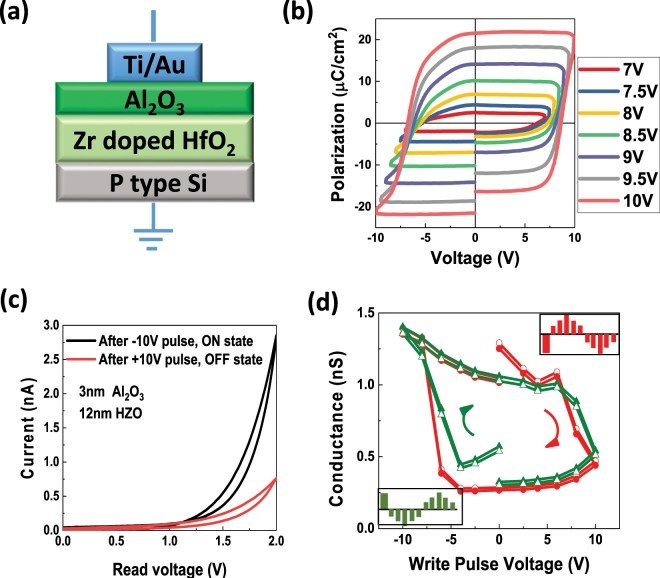
Polarization and hysteresis loop in FTJs based on Ti/Al_2_O_3_/HZO/p-Si. (**a**) Illustration of FTJs based on Al_2_O_3_/HZO/Si. (**b**) Polarization-Voltage (P-V) loops of an FTJ with 3 nm Al_2_O_3_/12 nm HZO. (**c**) The DC IV curves of the FTJs measured after −10 V and +10 V program pulses. (**d**) Hysteresis loops of the tunneling conductance of FTJs based on Ti/Al_2_O_3_/HZO/p-Si at 2V read voltage. The pulse trains are shown schematically in the insets. The red conductance lines correspond to the red pulse sequence shown in the upper right corner, while the green conductance lines correspond to the green pulse sequence shown in the lower left corner. The orange circle between two pulses means dc IV measurement for reading the conductance of the FTJ. The solid symbols are the conductance taken from the forward dc IV sweep (0 V to 2 V), while the open symbols are the conductance taken from the backward dc IV sweep (2 V to 0 V).

The FTJs based on Al_2_O_3_/HZO stacks were fabricated on highly-doped Si substrates using atomic layer deposition (ALD). The device structure is illustrated in Fig. 1a. We alternately stacked HfO_2_ and ZrO_2_ layers by using the Hf precursor [tetrakis(dimethylamido)hafnium] and Zr precursor [tetrakis(dimethylamido)zirconium]. Then, we deposited Al_2_O_3_ by using Al precursor [trimethylaluminium]. The encapsulated HZO films were then annealed in a rapid thermal annealing (RTA) system. Ti/Au electrodes (10/40 nm) were deposited by e-beam evaporation. The polarization of the doped HfO_2_ is measured using the positive-up-negative-down (PUND) method. The polarization-voltage (P-V) loops are shown in Fig. 1b. The remanent polarization in the FTJs can reach 21 µC/cm^2^ and is tunable by the pulse amplitude. The tunneling currents as a function of voltage after positive and negative program pulses are shown in Fig. 1c. The tunneling conductance at 2 V is plotted as function of program pulse value, shown in Fig. 1d. The insets show the pulse sequences. Non-volatile resistance switching is clearly observed. The positive pulses set the device to the low conductance OFF-state, whereas negative pulses switch the device to the high conductance ON-state. Notice that the two hysteresis loops (red and green) measured using two different pulse sequences are nearly identical, indicating that the TER hysteresis is reproducible.

**Figure 2:**
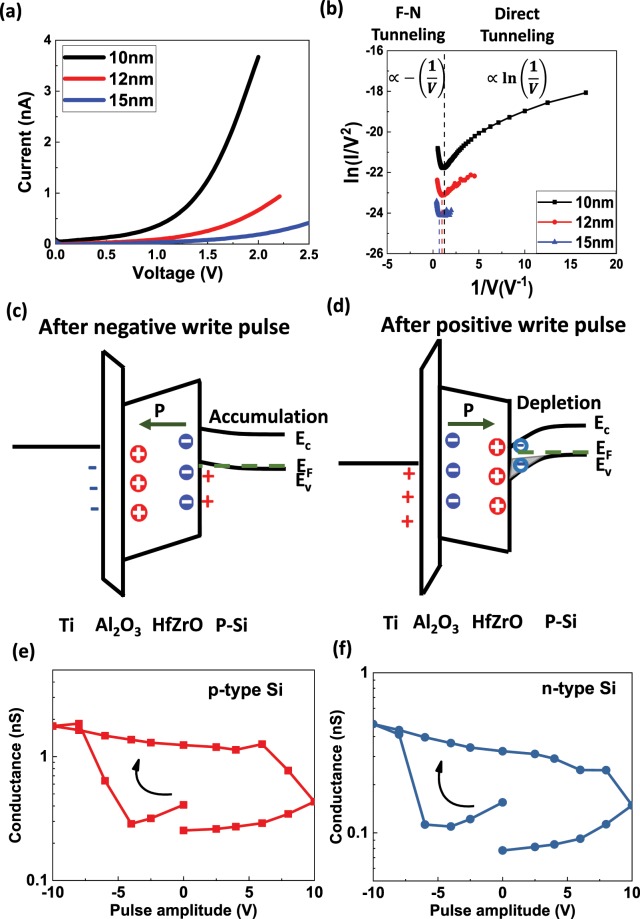
Mechanism of the hysteresis loops in FTJs based on Ti/Al_2_O_3_/HZO/p-Si. (**a**) Tunneling current as a function of voltage for FTJs with various HZO thicknesses at ON-state. (**b**) *In*(*I*/*V*^2^) as a function of 1/*V* for the data shown in (**a**). (**c,d**) Energy band diagrams of the FTJ after negative and positive write pulses respectively. (**e**,**f**) Hysteresis loops of the tunneling conductance at 2 V of FTJs based on Ti/Al_2_O_3_/HZO on p-type and n-type Si substrates respectively.

These TER hysteresis loop can be explained by the following analysis. Figure 2a shows the tunneling current as a function of applied voltage for FTJs based on metal/Al_2_O_3_/HZO/Si structure with various HZO thicknesses (10 nm, 12 nm and 15 nm) at ON-state. We plotted *In*(*I/V*^2^) versus 1/*V*, where *I* is the ON-state tunneling current and *V* is the applied voltage, shown in Fig. 2b. At high bias, *In*(*I*/*V*^2^) decreases linearly with 1/*V*, indicating that Fowler-Nordheim (F-N) tunneling dominates. At low bias, *In*(*I/V*^2^) increases logarithmically with 1/*V*, which is consistent with direct tunneling^38^. For the TER hysteresis loop shown in Fig. 1d, the read voltage is 2 V, which is in the F-N tunneling regime. The energy diagrams of the FTJs after positive and negative program pulses are shown in Fig. 2c,d respectively. When a negative pulse is applied on the top electrode, the polarization in HZO points to the electrode, which will induce a band tilt in HZO. In addition, the screening charge drives p-type silicon into accumulation, which will reduce tunneling width and increase the tunneling current. A positive read voltage will further enhance the band tilt in HZO and increase the F-N tunneling. These two factors will lead to high conductance in the FTJ. To further understand the impact of these two factors, we fabricated FTJs with p-type and n–type substrates and compared their TER hysteresis, shown in Fig. 2e,f. The hysteresis loops of these two devices are both clockwise with similar TER ratios. If the substrate depletion/accumulation is the dominant factor for the hysteresis loop, we would expect that the direction of the hysteresis loop will be opposite for FTJs with n- and p-type substrates. This result confirms that the polarization induced F-N tunneling barrier change is the main source for the hysteresis loop observed in these FTJs.

**Figure 3:**
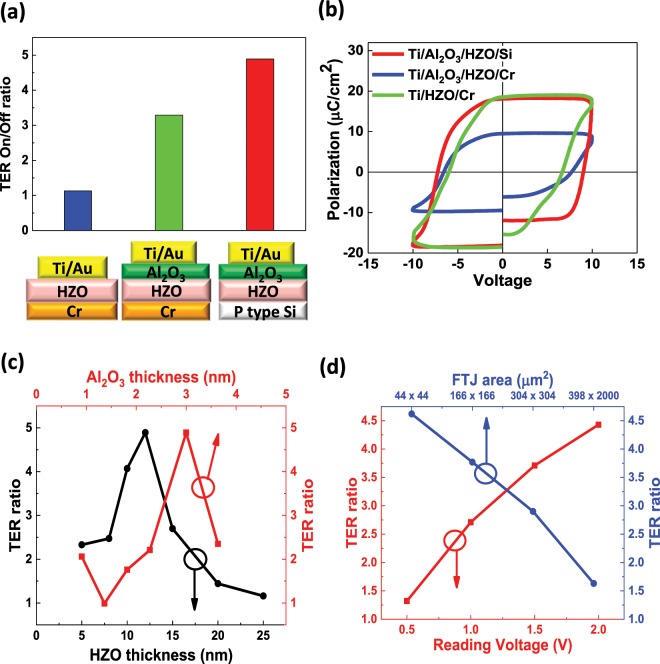
FTJs based on various structures. (**a**) TER ratios and (**b**) P-V loops of the FTJs based on Ti/HZO/Cr, Ti/Al_2_O_3_/HZO/Cr, and Ti/Al_2_O_3_/HZO/p-Si structures. (**c**) TER ratio as a function of Al_2_O_3_ thickness (top x axis) and HZO thickness (bottom x axis). (**d**) TER ratio as a function of FTJ area (top x axis) and read voltage (bottom x axis).

The ultra-thin Al_2_O_3_ tunneling layer plays an important role in these FTJs. As shown in Fig. 3a, the TER ratio of the FTJ based on metal/Al_2_O_3_/HZO/Si is much higher than that on metal/HZO/metal and metal/Al_2_O_3_/HZO/metal structures. Here, the interfacial Al_2_O_3_ layer facilitates the tuning of the band offset between HZO and metal, which can effectively enhance the TER ratio. Silicon substrate is also an important component in the FTJs. As shown in Fig. 3b, the remanent polarization of the FTJ based on a silicon substrate is much higher than that on Cr metal substrate, which explains the higher TER ratio observed in the FTJ based on silicon structure as compared to that on a metal substrate.

For FTJs based on metal/Al_2_O_3_/HZO/Si structure, the optimal Al_2_O_3_ thickness is ~3 nm, shown in Fig. 3c. An ultra-thin Al_2_O_3_ layer cannot provide enough tunability on the band offset, while too thick an Al_2_O_3_ layer can impede the tunneling current. The optimal HZO thickness is 10–12 nm, where ultrathin HZO does not have sufficient polarization, while thick HZO is difficult to tunnel through. TER ratios can be enhanced by using a smaller device area due to increased homogeneity and by using higher read voltage due to enhanced F-N tunneling (Fig. 3d).

**Figure 4:**
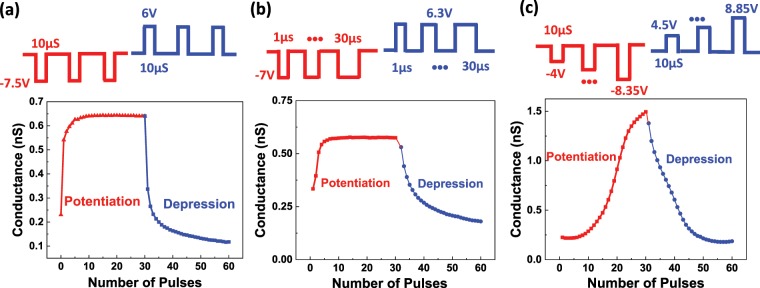
Tunneling conductance of FTJs as a function of pulse numbers. (**a**) Scheme 1: constant pulse amplitude and width. (**b**) Scheme 2: modulation of pulse width. (**c**) Scheme 3: modulation of pulse amplitude.

For the synaptic application, the tunneling resistance needs to be continuously tunable to emulate biological synapses. Figure 4a–c show the TER as a function of the pulse number with various pulse schemes. Scheme 1 consists of identical pulses with constant pulse amplitude and width. Scheme 2 consists of pulses with increasing pulse width while keeping the amplitude of the pulse constant. The pusle width for the *nth* pulse is ${t}_{n}={t}_{1}+(n-1)\varDelta t$, where *t*_1_ is the width of the first pulse and Δ*t* is the increment of pulse width between two consequtive pulses. Scheme 3 consists of pulses with increasing pulse amplitude while keeping the pulse width constant. In all three cases, the FTJ conductance increases with the number of potentiation pulses and decreases with the number of depression pulses, indicating that the synaptic weight is continuously tunable. Among the three schemes, pulse amplitude modulation shows the best performance in terms of discrete multilevel states, linearity, and symmetry. The analog behavior due to partial polarization switching observed in these devices indicates the high potential of ferroelectric FTJs in neuromorphic application.

**Figure 5:**
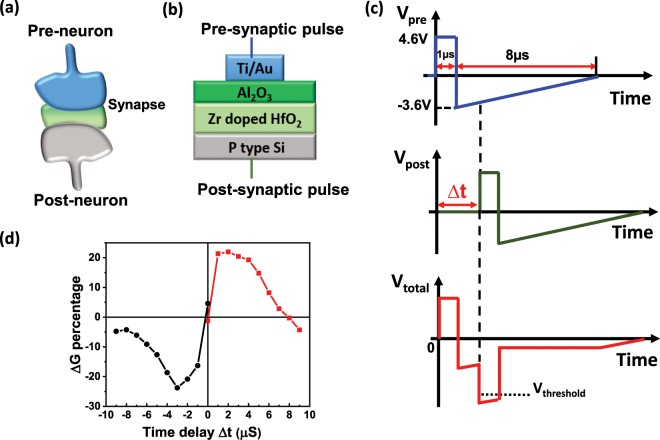
STDP of FTJs based on Al_2_O_3_/HZO stack. (**a**) Sketch of pre- and post-neurons connected by a synapse. (**b**) Schematic of HZO FTJ. (**c**) The waveforms of the pre-synaptic pulse *V*_*pre*_, post-synaptic pulse *V*_*post*_ and the total voltage *V*_*total*_ = *V*_*pre*_ − *V*_*post*_ on a FTJ. The pre-synaptic pulse is applied on the top gate, while the post-synaptic pulse is applied on the back gate of the FTJ. (**d**) Measurement of STDP in a FTJ based on Al_2_O_3_/HZO stack.

In biologic synapses, STDP is considered an important mechanism for memory and learning. We characterized the STDP characteristics of the FTJ synapses using pairs of pulses, emulating the spikes from pre- and post-neurons as illustrated in Fig. 5c. When the pre- and post-synaptic pulses reach the FTJs with a time delay Δ*t*, their superposition produces the waveforms *V*_*total*_ = *V*_*pre*_ − *V*_*post*_. If the resulting combined waveform momentarily exceeds the threshold voltage, it will lead to an increase or a decrease of the FTJ conductance, depending on the sign of Δ*t*. Figure 5d shows the measured conductance change as a function of time delay between pre- and post-synaptic pulses Δ*t* in a FTJ based on Al_2_O_3_/HZO stack. Here the conductance change percentage is defined as $\% \Delta G=\frac{{G}_{f}-{G}_{i}}{\min ({G}_{i},{G}_{f})}$, where *G*_*i*_ is the initial conductance before applying the a pair of pulses, *G*_*f*_ is the final conductance after applying a pair of pulses and min(*G*_*i*_, *G*_*f*_) is the minimum of *G*_*i*_ and *G*_*f*_^39,40^. As can be seen in Fig. 5d, the conductance inceases when Δ*t* > 0, while decreases when Δ*t* < 0. Close-timed pulses leads to a large conductance change, whereas long delays between two pulses leave the FTJ unchanged. These phenomena can be understood through the waveform of the total voltage *V*_*total*_. If the post-synaptic pulse arrives after the pre-synaptic pulse (Δ*t* > 0), the peak total voltage is negative, which will increase the conductance of the FTJ (the synapse is strengthened), as illustrated in supplementary information (Fig. S2). When the post-synaptic pulses come before the pre-synaptic pulse (Δ*t* < 0), the peak total voltage is positive, thus the conductance of FTJ decreases (the synapse is weakened). The absolute value of $|\varDelta t|$ can influence the amplitude of the conductance change. As illustrated in Fig. S3, small time gap $|\varDelta t|$ results in large amplitude of peak voltage $|{V}_{total}|$, which leads to dramatic change of conductance in FTJ. These results indicate that the FTJs based on Al_2_O_3_/HZO stacks can mimick the STDP function of biologic synapses.

**Figure 6:**
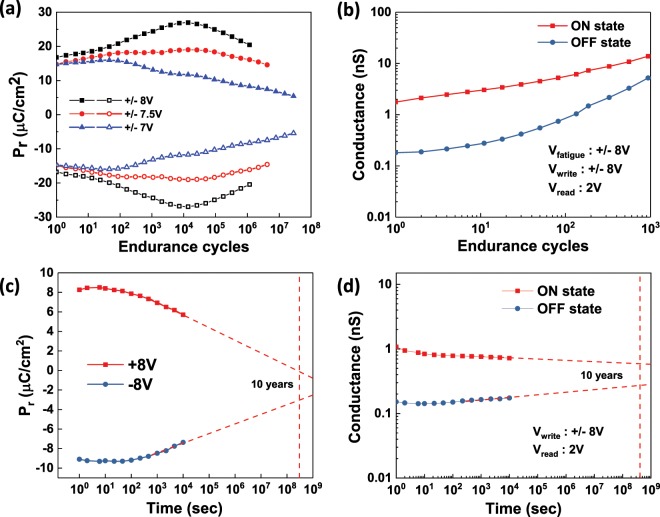
The reliability of the FTJs based on Al_2_O_3_/HZO/Si structure. (**a**) Remanent polarization as a function of endurance cycles measured at different cycling voltages: ±7 V, ±7.5 V, and ±8 V. The amplitudes of the cycling and the PUND read pulses are the same. The pulse width is 10 µs for all cases. (**b**) Tunneling conductance at ON- and OFF-states as a function of endurance cycles. The amplitude of the cycling and program pulse is 8 V. The tunneling conductance is extracted at 2 V from the dc IV measurement. (**c**) Remanent polarization as a function of retention time. The amplitudes of the program and the PUND read pulses are 8 V. The program pulse width is 10 µs. The read pulse width is 100 ns. (**d**) Tunneling conductance at ON- and OFF-states as a function of retention time. The program pulse amplitude is 8 V and the tunneling conductance is extracted at 2V from the dc IV measurement.

We further investigated the reliability of the FTJs based on the Al_2_O_3_/HZO/Si structure. Figure 6a shows the remanent polarization as a function of the cycle numbers. At the ±7 V cycling voltage, the device shows the characteristic fatigue behavior and the endurance is over 10^7^ cycles. At a higher cycling voltage (±8 V), a wake-up effect at early stage and a fatigue effect at the late stage followed by a hard breakdown were observed, The endurance is ~10^6^ cycles at ±8 V cycling voltage. The evolution of the tunneling conductance in the ON- and OFF-states with cycling numbers is shown in Fig. 6b. Both the ON- and OFF-conductance increases with the cycle numbers due to the stress-induced leakage current, which can be attributed to interface and bulk traps formed in the dielectric during the high voltage pulses. A TER ratio of ~2.65 remains after 10^3^ cycles at ± 8 V cycling voltage. The retention of the FTJs is also characterized. Figure 6c shows the remanent polarization as a function of retention time. The remanent polarization reduces gradually with time due to the depolarization field, which can be attributed to the incomplete charge compensation at the Al_2_O_3_/HZO and HZO/Si interfaces. As a result, the tunneling conductance contrast between ON- and OFF-states reduces with time accordingly, shown in Fig. 6d. At room temperature, a TER ratio of ~2.64 remains after extrapolation to 10 years.

## Conclusion

In conclusion, we demonstrated a new type of FTJ based on Al_2_O_3_/HZO/Si structure for neurosynaptic applications. We found that an Al_2_O_3_ interfacial layer and the silicon substrate can significantly enhance the TER ratio. The ferroelectricity in Zr-doped HfO_2_ can be as high as 21 µC/cm^2^ and is tunable by the program pulse amplitude and duration, which is critical for synaptic applications. We found that the current in these devices is dominated by direct tunneling at low bias and by F-N tunneling at high bias. The polarization switching in HZO can alter the tunneling barrier height and lead to different tunneling resistance states. The TER ratios of the FTJs are influenced by the thickness of Al_2_O_3_ and HZO, the area of the device, and the read voltage. These FTJs show tunable multilevel resistance states, linear potentiation/depression characteristics, and strong STDP behavior, which are the critical traits for artificial synapses. The retention of the FTJs is longer than 10-years at room temperature and the FTJs can endure over 10^7^ cycles at 7 V program voltage. These novel FTJs will have high potential in both energy-efficient non-volatile memories and artificial neural network applications.

## Supplementary information

Supplementary information.
