# Supplementary material for: Ferroelectric Tunneling Junctions Based on Aluminum Oxide/ Zirconium-Doped Hafnium Oxide for Neuromorphic Computing
Source: Sci Rep. 2019 Dec 31;9:20383. doi: 10.1038/s41598-019-56816-x (PMC6938512; doi:10.1038/s41598-019-56816-x)
Supplement: Supplementary file 1 — Supplementary information. [file 41598_2019_56816_MOESM1_ESM.pdf]

Supplementary Information

Ferroelectric Tunneling Junctions Based on Aluminum Oxide/ Zirconium-Doped Hafnium Oxide for Neuromorphic Computing

Author: Hojoon Ryu[1], Haonan Wu[1], Fubo Rao[2], and Wenjuan Zhu[1]

[1]Affilliation: Department of Electrical and Computer Engineering, University of Illinois at Urbana-Champaign, Urbana, IL 61801, USA

[2]Affilliation: Materials Research Laboratory, University of Illinois at Urbana-Champaign, Urbana, IL 61801, USA

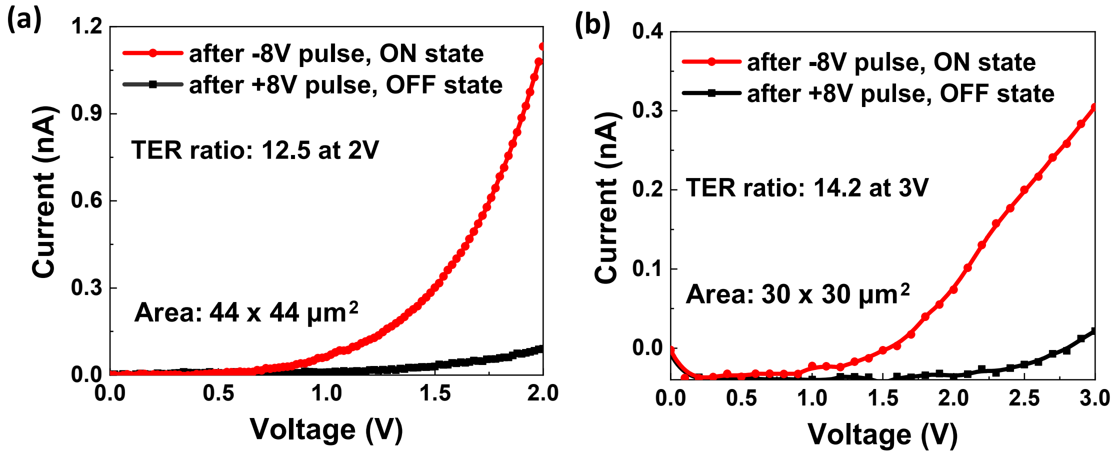

Figure S1. The DC IV curves of the FTJs based on Ti/Al_2O_3/HZO/p-Si measured after ±8 V program pulses. (a) The TER ratio of the FTJ with $44 \times 44\mu m^2$ area is 12.5 at 2V. (b) The TER ratio of the FTJ with $30 \times 30\mu m^2$ area is 14.2 at 3V.

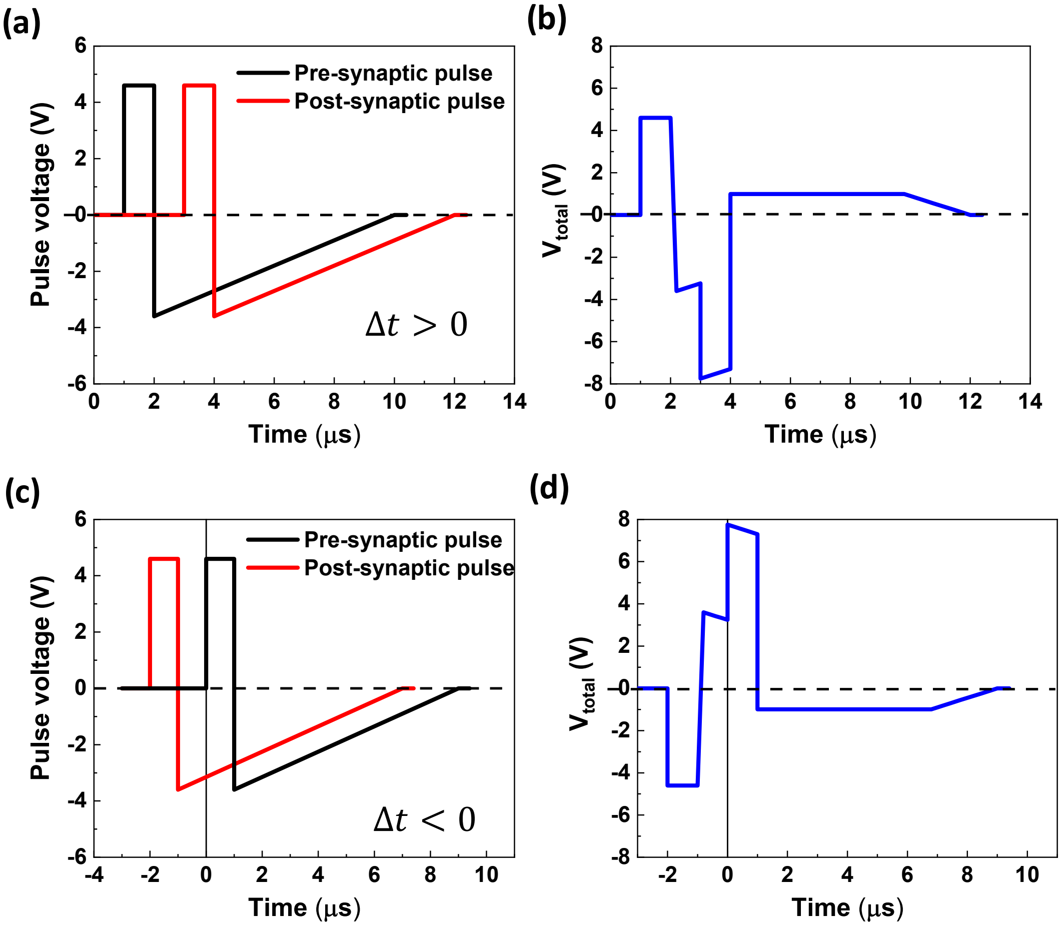

Figure S2. Impact of the sign of Δt, which is the time delay between pre- and post-synaptic pulses, on the resulting combined waveform V_{total}. (a) The waveform of pre- and post-synaptic pulses with $\Delta t = 2\mu s$ and $t_0 = 1\mu s$. The resulting combine waveform is shown in (b). The largest amplitude of V_{total} is reached at $3\mu s$ and the peak V_{total} is negative. (c) The waveform of pre- and post-synaptic pulses with $\Delta t = -2\mu s$ and $t_0 = 1\mu s$. The resulting combine waveform is shown in (d). The largest amplitude of V_{total} is reached at $3\mu s$ and the peak V_{total} is positive.

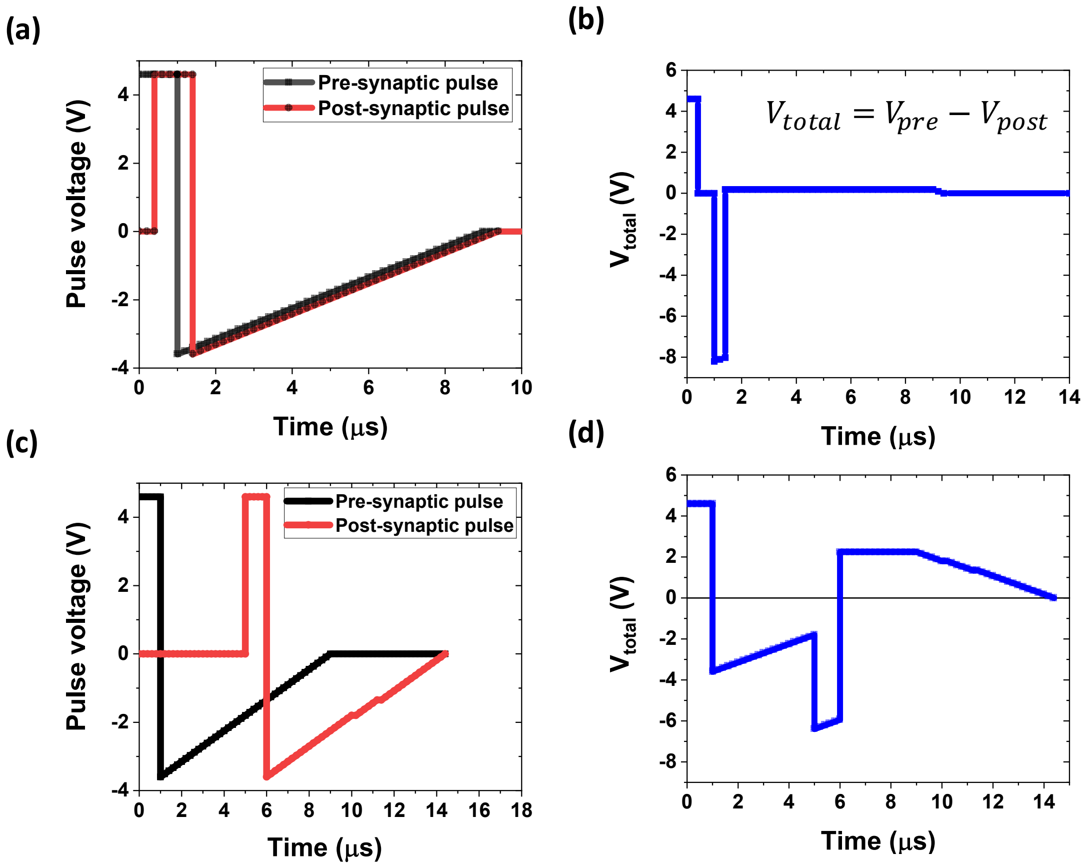

Figure S3. Impact of the amplitude of Δt, which is the time delay between pre- and post-synaptic pulses, on the resulting combined waveform V_{total}. (a) The waveform of pre- and post-synaptic pulses with $\Delta t = 0.4\mu s$ and $t_0 = 1\mu s$. The resulting combine waveform is shown in (b). The largest amplitude of V_{total} is 8.2 V. (c) The waveform of pre- and post-synaptic pulses with $\Delta t = 5\mu s$ and $t_0 = 1\mu s$. The resulting combine waveform is shown in (d). The largest amplitude of V_{total} is 6.4 V.
